# CNS Hypomyelination Disrupts Axonal Conduction and Behavior in Larval Zebrafish

**DOI:** 10.1523/JNEUROSCI.0842-21.2021

**Published:** 2021-11-03

**Authors:** M. E. Madden, D. Suminaite, E. Ortiz, J. J. Early, S. Koudelka, M. R. Livesey, I. H. Bianco, M. Granato, D. A. Lyons

**Affiliations:** ^1^Centre for Discovery Brain Sciences, University of Edinburgh, Edinburgh EH16 4SB, United Kingdom; ^2^Perelman School of Medicine, University of Pennsylvania, Philadelphia, Pennsylvania 19104; ^3^Sheffield Institute for Translational Neuroscience, Department of Neuroscience, The University of Sheffield, Sheffield S10 2HQ, United Kingdom; ^4^Neuroscience, Physiology and Pharmacology, University College London, London WC1E 6BT, United Kingdom

**Keywords:** circuit function, electrophysiology, myelin, *myrf*, oligodendrocyte, zebrafish

## Abstract

Myelination is essential for central nervous system (CNS) formation, health and function. As a model organism, larval zebrafish have been extensively employed to investigate the molecular and cellular basis of CNS myelination, because of their genetic tractability and suitability for non-invasive live cell imaging. However, it has not been assessed to what extent CNS myelination affects neural circuit function in zebrafish larvae, prohibiting the integration of molecular and cellular analyses of myelination with concomitant network maturation. To test whether larval zebrafish might serve as a suitable platform with which to study the effects of CNS myelination and its dysregulation on circuit function, we generated zebrafish myelin regulatory factor (*myrf*) mutants with CNS-specific hypomyelination and investigated how this affected their axonal conduction properties and behavior. We found that *myrf* mutant larvae exhibited increased latency to perform startle responses following defined acoustic stimuli. Furthermore, we found that hypomyelinated animals often selected an impaired response to acoustic stimuli, exhibiting a bias toward reorientation behavior instead of the stimulus-appropriate startle response. To begin to study how myelination affected the underlying circuitry, we established electrophysiological protocols to assess various conduction properties along single axons. We found that the hypomyelinated *myrf* mutants exhibited reduced action potential conduction velocity and an impaired ability to sustain high-frequency action potential firing. This study indicates that larval zebrafish can be used to bridge molecular and cellular investigation of CNS myelination with multiscale assessment of neural circuit function.

**SIGNIFICANCE STATEMENT** Myelination of CNS axons is essential for their health and function, and it is now clear that myelination is a dynamic life-long process subject to modulation by neuronal activity. However, it remains unclear precisely how changes to myelination affects animal behavior and underlying action potential conduction along axons in intact neural circuits. In recent years, zebrafish have been employed to study cellular and molecular mechanisms of myelination, because of their relatively simple, optically transparent, experimentally tractable vertebrate nervous system. Here we find that changes to myelination alter the behavior of young zebrafish and action potential conduction along individual axons, providing a platform to integrate molecular, cellular, and circuit level analyses of myelination using this model.

## Introduction

Myelination is a well-characterized regulator of axonal health and function. In recent years, it has become clear that myelination in the CNS is dynamically regulated over time, including by neuronal activity, leading to the view that activity-regulated myelination might represent a form of functional plasticity (Fields, 2015). Furthermore, disruption to myelin is observed in numerous diseases of the CNS, and its regulation may represent a viable therapeutic strategy. Indeed, major insights have emerged from studies in multiple systems into the cellular and molecular mechanisms of CNS myelination, its regulation by neuronal activity, and its disruption in disease (Nave and Werner, 2014; Mount and Monje, 2017; Almeida, 2018; Gibson et al., 2018). In parallel, an increasing number of studies indicate that the generation of new oligodendrocytes (McKenzie et al., 2014; Geraghty et al., 2019; Pan et al., 2020; Steadman et al., 2020; Wang et al., 2020), and the degree of myelination (Makinodan et al., 2012; Sampaio-Baptista et al., 2013; Liu et al., 2016; Bonnefil et al., 2019), are important for distinct behaviors. However, how dynamic regulation of myelination, or disruption to myelin per se, actually affects the activity of circuits underlying these behaviors remains much less clear. This is partly because of the difficulty in visualizing changes to myelination along single axons over time in the mammalian brain while concomitantly assessing their conduction properties and, in turn, evaluating how alteration to conduction affects neural circuit function.

Zebrafish are well established as a model organism for the study of myelination. The small size and transparency of their larvae, in combination with their genetic tractability and established transgenic tools, allows the assessment of myelin made by individual oligodendrocytes and along single axons *in vivo* (Koudelka et al., 2016; Auer et al., 2018). Together, these features have facilitated many discoveries into the molecular and cellular mechanisms of myelination in this model (Preston and Macklin, 2015). Despite this progress, it remains unknown how CNS myelination affects the function of individual axons, neural circuits, or the behavior of larval zebrafish, and thus it is not clear whether integrated multiscale assessments of CNS myelination from molecule through circuit can be performed in this model. However, it is now clear that larval zebrafish exhibit a diverse repertoire of experimentally tractable innate and stereotypical locomotor behaviors (Marques et al., 2018), many of which are mediated by reticulospinal (RS) neurons, a diverse set of neurons of the midbrain and hindbrain that process multimodal sensory information, and project descending axons to the spinal cord to coordinate specific motor outputs (Metcalfe et al., 1986; Gahtan and O'Malley, 2003). Intriguingly, RS axons are the first to be myelinated in the zebrafish CNS and exhibit activity-regulated myelination at larval stages (Almeida et al., 2011; Hines et al., 2015; Koudelka et al., 2016), implying that regulation of their myelination might influence circuit function in early larvae. *In vivo* electrophysiological recordings from subsets of individual RS neurons are feasible (Saint-Amant and Drapeau, 2003; Tanimoto et al., 2009; Roy and Ali, 2013), which in principle permits direct measurement of myelinated axon conduction properties underlying behavior. However, how disruption to CNS myelination affects the behavior or axonal conduction properties of larval zebrafish remains to be investigated.

In this study, we set out to investigate whether changes to CNS myelination can be detected in behavior and in the conduction properties of single axons in zebrafish larvae. To achieve this, we created a myelin gene regulatory factor (*myrf*) mutant line, which exhibits severe CNS hypomyelination. Using this mutant, we demonstrate that both behavioral and electrophysiological consequences of hypomyelination are indeed detectable in the relevant circuitry *in vivo*, providing proof of principle that integrated analysis is feasible in this model organism, offering a framework for future investigations.

## Materials and Methods

### Zebrafish maintenance

Zebrafish were raised and maintained under standard conditions in the BVS Aquatics Facility in the Queen's Medical Research Institute, University of Edinburgh. Adult and larval animals were maintained on a 14/10 h light/dark cycle. Embryos were stored in 10 mm HEPES-buffered E3 embryo medium or conditioned aquarium water with 0.000001% methylene blue at 28.5°C. All experiments were performed under the project license 70/8436 with approval from the United Kingdom Home Office. The *myrf^ue70^* line was maintained in a Tupfel Long Fin (TL) wild-type background. Within this article, Tg denotes a stable, germline inserted transgenic line.

### Transgenic and mutant lines

The *myrf^ue70^* mutant line was established during this study and is described in this article. The following transgenic lines were also used in this study: Tg(mbp:eGFP-CAAX) (Almeida et al., 2011; Mensch et al., 2015) and Tg(mbp:nls-eGFP) (Karttunen et al., 2017).

### Generation of myrf^ue70^ mutants

A freely available guide selection tool (http://crispr.mit.edu) was used to select sgRNA sequences against the second exon of the zebrafish *myrf* gene. sgRNA (target sequence CATTGACACCAGTATCCTGG) was synthesized using DNA template oligomers (5′-AAAGCACCGACTCGGTGCCACTTTTTCAAGTTGATAACGGACTAGCCTTATTTTAACTTGCTATTTCTAGCTCTAAAAC**CCAGGATACTGGTGTCAATG**CTATAGTGAGTCGTATTACGC-3′; Integrated DNA Technologies) consisting of DNA coding for the T7 promotor, DNA recognition sequence (sgRNA variable region), indicated in bold and the sgRNA scaffold. sgRNA synthesis was performed using Ambion MEGAshortscript T7 Transcription kit (Thermo Fisher Scientific) using the synthesized DNA oligomers as template. Transcribed sgRNA was purified using Ambion MEGAclear kit (Thermo Fisher Scientific). The expression vector for Cas9 protein, pCS2-nCas9 (Addgene plasmid #47929; Jao et al., 2013), was used to transcribe Cas9 mRNA using the mMESSAGE mMachine SP6 kit (Thermo fisher Scientific) and purified using an RNeasy mini kit (QIAGEN). Injection solutions were prepared to a final concentration of 300 ng/µl nCas9 mRNA and 10 ng/µl sgRNA in nuclease free water and 0.05% phenol red (Sigma-Aldrich). Wild-type embryos were injected at the single or two-cell stage with 1.5-nl injection solution. Injected F0 animals were raised to adulthood and outcrossed to wild-type animals to create F1 offspring. Clutches of F1 offspring were raised to adulthood and genotyped to identify heterozygous carriers of function disrupting mutant alleles. *myrf^ue70^* refers to a specific allelic mutation consisting of the deletion of two cytosine nucleotides and insertion of a single adenine nucleotide (wild-type sequence: 5′-CCAGTAT**CC**TGGAGGAATA-3′; *myrf^ue70^* mutant allele: 5′-CCAGTAT**A**TGGAGGAATA-3′).

### Genotyping

Tissue was genotyped using primers myrf-f (5′-AACTGTGCGTAGGAACACGATA-3′) and myrf-r (5′-TGGACCTCCGTGAAACAACTG-3′) in a standard PCR. The PCR product was digested using restriction enzyme PspGI (New England Biolabs), which cleaves wild-type product into 131- and 157-bp fragments. The mutant product remains uncut as the *myrf^ue70^* allele contains a frameshifting indel which abolishes the PspGI cutting site. PCR products were visualized on a 2% gel following gel electrophoresis. All analyses were performed blinded to genotype.

### Quantitative RT-PCR

Total RNA was extracted from whole brains of adult *myrf^ue70^* wild-type and homozygous siblings using a modified TRIzol RNA extraction protocol (TRIzol reagent, Thermo Fisher Scientific). RNA concentration and integrity were assessed using a nanodrop spectrophotometer (NanoDrop One^c^, Thermo Fisher Scientific). RNA clean-up was performed if necessary. cDNA synthesis was performed using Accuscript Hi Fidelity First Strand Synthesis kit (Agilent). The amount of RNA entered into the reaction was normalized between samples. Primers mbp-f (5′-ACAGAGACCCCACCACTCTT-3′) and mbp-r (5′-TCCCAGGCCCAATAGTTCTC-3′) were used to amplify mbp transcripts within a qPCR (Brilliant III Ultra-fast SYBR Green qPCR Master Mix, Agilent). Transcript levels were detected using Roche Light Cycler 96 (Roche Life Science) with the following amplification protocol: preincubation 95° for 180 s, two-step amplification 40 cycles: 95° for 10 s then 60° for 20 s, followed by high-resolution melting. Each sample was run in triplicate. Housekeeping gene ef1a was used as a reference gene, using primers ef1a-f (5′-TGGTACTTCTCAGGCTGACT-3′) and ef1a-r (5′-TGACTCCAACGATCAGCTGT-3′). The δ-δ CT method was used to quantify expression levels. All values were normalized to wild types to provide the relative expression of the gene of interest.

### Transmission electron microscopy (TEM)

Larval tissue was prepared for TEM using the microwave fixation protocol as previously described (Czopka and Lyons, 2011; Karttunen et al., 2017). For adult tissue, adult zebrafish were terminally anaesthetized in tricaine and perfused intracardially with PBS followed by primary fixative solution (4% paraformaldehyde, 2.5% glutaraldehyde, and 0.1 m sodium cacodylate; Sigma-Aldrich). Adults were subsequently incubated in fresh primary fixative solution for 24 h at 4°C. Spinal cords were dissected and processed using the microwave fixation protocol described for larval tissue. TEM images were obtained using a JEOL JEM-1400 Plus Electron Microscope. Image magnification ranged from 11,200× to 17,000× magnification for larval spinal cords, and 1,700× for adult spinal cord.

### Single-cell labeling

Fertilized eggs from *myrf^ue70^* heterozygous adult in-crosses were microinjected between the single and four-cell stage with 10 ng/µl plasmid DNA encoding mbp:mCherry-CAAX (Mensch et al., 2015) and 25 ng/µl tol2 transposase mRNA in nuclease free water with 10% phenol red. Animals were screened at four days postfertilization (dpf) for mosaically labeled oligodendrocytes and subsequently imaged. Isolated single cells from any level in the dorsal spinal cord were imaged. Images were obtained in 4 and 6 dpf larvae.

### Live imaging

Larvae were anaesthetized in tricaine/MS-222 (ethyl 3-aminobenzoate methanesulfonate salt, Sigma-Aldrich) in HEPES-buffered E3 embryo medium and embedded in 1.3–1.5% low melting point agarose (Invitrogen). All fluorescent images were acquired using a Zeiss LSM 880 confocal microscope with a 20× objective (Zeiss Plan-Apochromat 20× dry, NA = 0.8, Carl Zeiss Microscopy). Z-stacks were obtained through the entire single cell, axon, or spinal cord according to each experiment. For time course imaging, a single oligodendrocyte was imaged at 4 dpf. Larvae were then extracted from agarose gel, recovered in embryo medium and maintained with daily feeds and water exchange until imaging of the same cell was repeated at 6 dpf. For automated imaging of the entire spinal cord and peripheral nervous system (PNS), vertebrate automated screening technology (VAST) was used as described previously (Early et al., 2018). Briefly, larvae are arrayed into individual wells of a 96-well plate containing MS-222-treated HEPES-buffered E3 embryo media. Fish are loaded and oriented for imaging using a Large Particle (LP) Sampler and VAST BioImager system (Union Biometrica Inc) fitted with a 600-µm capillary tube. Embryos are automatically loaded into the capillary, positioned and imaged using an AxioCam 506m CCD Camera, a CSU-X1 spinning disk confocal scanner, a 527/54 + 645/60 nm double bandpass emission filter, 1.6× C-Mount adapter, a PIFOC P-725.4CD piezo objective scanner, W-Plan-Apochromat 10 × 0.5 NA objective and an Axio Examiner D1. Z-stacks covering the depth of the capillary were acquired using a 4-µm z-interval, 3 × 3 binning and 60 ms exposure. Images were acquired using brightfield and the appropriate fluorescent channel. Following imaging, larvae were dispensed into a corresponding well of a 96-well collection plate and whole tissue retained for genotyping. Unless otherwise stated, all confocal images presented in this article represent a lateral view of the zebrafish spinal cord, with anterior to the left and posterior to the right, and dorsal/ventral at the top/bottom of the image, respectively. Within experiments, images were obtained using similar laser intensity and optical gain settings. All imaging was performed blinded to genotype.

### TEM image processing and analysis

TEM images were tiled using the automated photograph merge tool in Adobe Photoshop 2019. The number of ensheathed axons was counted in one hemi-spinal cord section per larva using the cell counter tool in FIJI ImageJ. Axon caliber is defined as the area of the axon within this article. Axonal area was calculated using the freehand line and measure tool in FIJI ImageJ. A g-ratio represents the ratio between the inner and outer diameter of the myelin sheath (i.e., a larger g-ratio values denotes a thinner myelin sheath). This calculation assumed perfect circularity of axons, which is not true to larval zebrafish TEM preparations. Thus, within these experiments g-ratio was calculated by dividing axonal area by the axonal + associated myelin area.

### VAST image processing and analysis

Images obtained using VAST were stitched and processed using FIJI ImageJ software (Schindelin et al., 2012) and custom macros (Early et al., 2018). Semi-automated oligodendrocyte counts were performed on the maximum intensity projection images (Early et al., 2018). Cell count values represent all oligodendrocytes in the spinal cord (dorsal and ventral tracts). Morphometric analysis of larval developmental features was performed on brightfield images. Measurements of ocular diameter, body length and swim bladder height were performed using the line and measure tool in FIJI ImageJ (National Institutes of Health).

### Single-cell image processing and analysis

Confocal z-stack images were processed using Zen software (Zeiss). Images were opened in FIJI ImageJ. Cells were included for analysis only if all myelin sheaths were distinguishable. Myelin sheath lengths were measured using the freehand line and measure tools. Myelin sheath number was equivalent to the number of measurements performed. Total myelin per cell was calculated as sum of all myelin sheath lengths per cell. Abnormal sheaths were defined as sheaths with abnormal elongation profiles, incomplete wrapping or myelin blebbing. For time course experiments, net growth or shrinkage of myelin sheaths was calculated as the average myelin sheath length at 6 dpf minus the average myelin sheath length at 4 dpf. Where possible, all myelin sheaths per cell were measured. In instances where measurement of all myelin sheaths was not possible (because of other cells coming into close proximity), only isolated myelin sheaths were analyzed at each time point. The number of fully retracted sheaths was recorded, and these sheaths were excluded from sheath growth analysis.

### Electrophysiology

Zebrafish were dissected as described previously in Roy and Ali (2013) to access the Mauthner neuron. In short, 6 dpf anaesthetized zebrafish were laid on their sides on a Sylgard dish and secured using tungsten pins through their notochords in a dissection solution containing the following: 134 mm NaCl, 2.9 mm KCl, 2.1 mm CaCl_2_, 1.2 mm MgCl_2_, 10 mm HEPES, 10 mm glucose, and 160 mg/ml tricaine, adjusted to pH 7.8 with NaOH. Their eyes as well as lower and upper jaws were removed using forceps to expose the ventral surface of the hindbrain, which was secured with an additional tungsten pin. The motor neurons in the anterior spinal cord were exposed as described by Wen and Brehm (2005). A dissecting tungsten pin was used to remove the skin and the muscle overlaying the motor neurons in a single segment. Following the dissection, zebrafish together with their recording chamber were moved to the rig and washed with extracellular solution containing the following: 134 mm NaCl, 2.9 mm KCl, 2.1 mm CaCl_2_, 1.2 mm MgCl_2_, 10 mm HEPES, and 10 mm glucose with 15 µm tubocurarine. The cells were visualized using an Olympus microscope capable of DIC using a 60×water immersion NA = 1 objective lens and Rolera Bolt Scientific camera with Q-Capture Pro 7 software. The stimulating electrode filled with extracellular solution was then positioned in the mid spinal cord lightly touching the exposed neurons underneath. Mauthner whole-cell recordings were performed with thick-walled borosilicate glass pipettes pulled to 6–10 MΩ. The internal solution contained the following: 25 mm K-gluconate, 15 mm KCl, 10 mm HEPES, 5 mm EGTA, 2 mm MgCl_2_, 0.4 mm Na_3_GTP, 2 mm Na_2_ATP, and 10 mm Na-phosphocreatine, adjusted to pH 7.4 with KOH. Upon formation of whole-cell patch clamp, 270 s-long recording was performed in the current-clamp configuration. Cell resting membrane potential was established as an average of the first 5 s of the recording if the cell did not fire during that time. To measure the conduction velocity along the Mauthner axon, the zebrafish were washed with recording solution containing the following: 134 mm NaCl, 2.9 mm KCl, 2.1 mm CaCl_2_, 1.2 mm MgCl_2_, 10 mm HEPES, and 10 mm glucose with addition of 50 µm AP5, 20 µm strychnine, 100 µm picrotoxin, and 50 µm CNQX. The antidromic Mauthner action potentials were recorded following field stimulation by the stimulating electrode connected to a DS2A Isolated Voltage Stimulator (Digitimer) in the spinal cord. Thirty consecutive action potentials were recoded every 5 s using Clampex 10.7 at 100 kHz sampling rate and filtered at 2 kHz using MultiClamp 700B. At the end of the recording, images of the zebrafish were obtained with a 4× objective and stitched using Adobe Photoshop. The resulting image was then transferred to FIJI and the distance between the stimulating and the recording electrodes was measured. The conduction velocity of action potential was calculated by dividing the distance between the electrodes by the latency from the stimulus artifact to the peak of action potential. Action potential latency and half-width were measured using homebuilt MATLAB scripts. For the analysis of action potential fidelity consecutive trains of 10 stimuli were delivered at 1, 10, 100, 300, 500, and 1000 Hz every 30 s. Recordings were made at 20 kHz sampling rate and filtered at 2 kHz. The number of action potentials was calculated using Clampfit 10.7 software and the % action potential success rate was calculated as a number of action potentials fired out of 10 and multiplied by 100.

### Behavioral assay

Analysis of startle behavior in 5–6 dpf *myrf^ue70^* mutant and wild-type larvae was performed as previously described (Burgess and Granato, 2007a; Wolman et al., 2011). Briefly, larvae were placed into individual wells of a 6 × 6 custom made acrylic testing plate containing E3 embryo media. A series of 10 acoustic stimuli (40.6 dB, 1000 Hz, 3 ms in duration) were delivered to the plate with an interstimulus interval of 20 s. Behavior was recorded using a high-speed camera (Photron Fastcam Mini UX) at 1000 frames/s. Analysis of recorded video footage was performed using FLOTE v2.0 tracking software (Burgess and Granato, 2007a). Larvae that responded to <70% of the stimuli were excluded from further kinematic analysis. Average behavioral latency was calculated as an average per larva over all behavioral responses. Short latency C-starts (SLC) and long latency C-starts (LLC) were defined by identifying a latency value (16 ms) separating the two peaks of the latency bimodal distribution in wild-type *myrf^ue70^* larvae. Behavioral latency, c-bend duration, initial turn angle, and angular velocity for SLC and LLC events were defined and analyzed as previously described (Burgess and Granato, 2007a).

### Experimental design and statistical analysis

Unless stated otherwise, all experiments were performed on 6 dpf larvae from adult heterozygous in-crosses. All subjects were the offspring of third generation, or younger, adults. The experimenter was blinded to the genotype of the larvae during all experiments and analysis. The sex of the animals was unknown as sex specification has not occurred at this stage of larval development. All graphs and statistical testing were performed using GraphPad Prism. All data were assessed for Gaussian distribution using a D'Agostino–Pearson omnibus normality test. Parametric continuous data were analyzed using a two-tailed unpaired Student's *t* test, or two-way ANOVA, according to the number of variables being compared. Non-parametric continuous data were analyzed using a Mann–Whitney test. If the number of values were too small to assess for normality, it was assumed that data were non-parametric. Results were considered statistically significant when *p* < 0.05. Within figures, *p* values are denoted as follows: non-significant (ns), *p*> 0.05, *p <0.05, **p <0.01, ***p < 0.001, ****p < 0.0001. Unless otherwise stated, all data were averaged per biological replicate (*N* represents number of larvae). Throughout the figures, error bars represent mean ± SD for parametric data, or median and interquartile range (IQR) for non-parametric data. Details of statistical tests, precise *p* and *n* values for each experiment are provided in the appropriate figure legends.

### Code accessibility

Custom written code to perform automated cell counts is available in a previous publication (Early et al., 2018). Code to interpret electrophysiological data are available at https://github.com/skotuke/Mauthner_analysis.

## Results

### Targeting myelin gene regulatory factor to create a larval zebrafish model of CNS-specific hypomyelination

**Figure 1.**
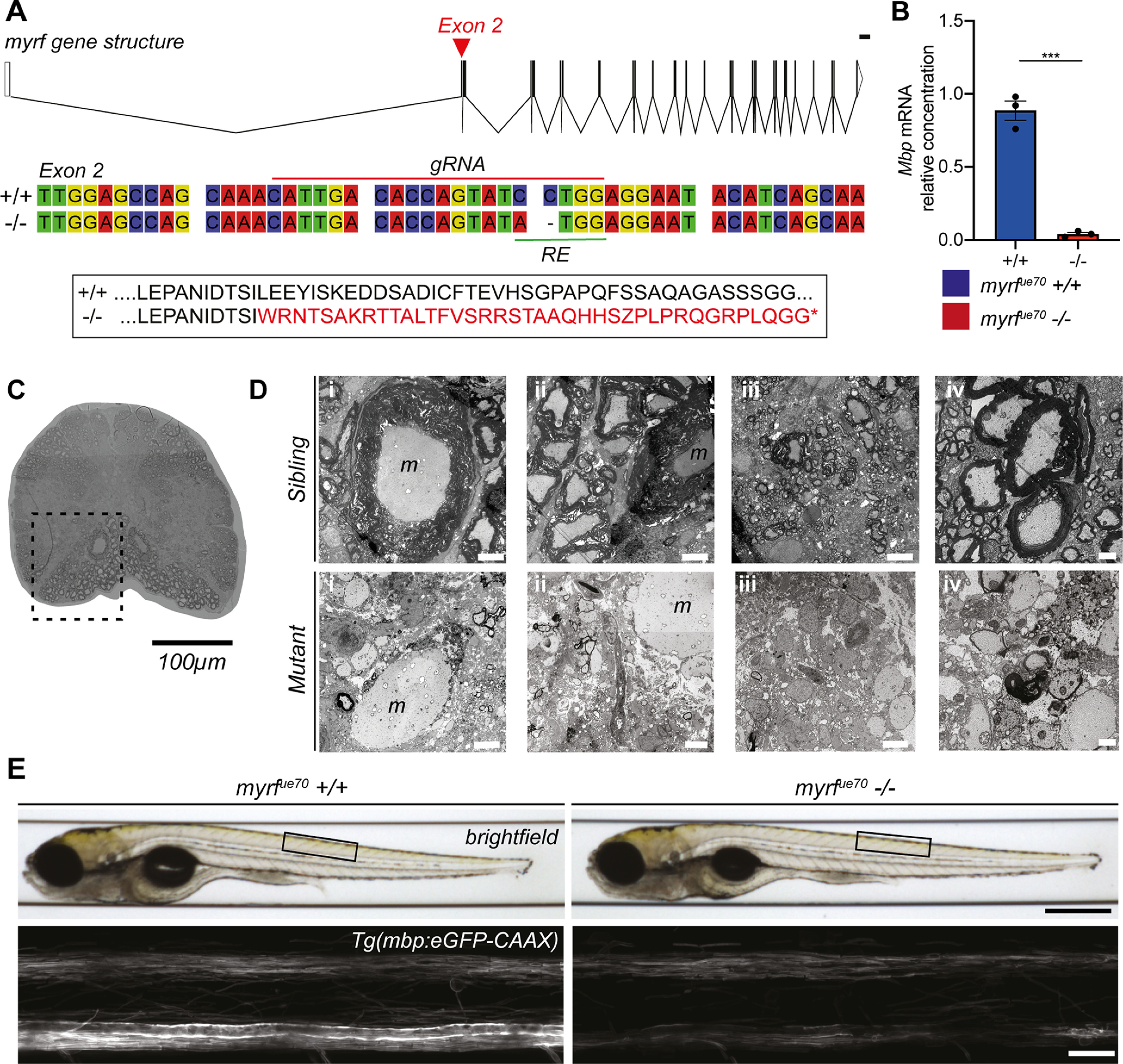
*myrf^ue70^* mutants display a gross reduction in the level of CNS myelination at the adult and larval stages. ***A***, top, myrf gene structure composed of 27 exons. Red arrowhead marks the location of the mutation in exon 2. Scale bar: 1000 bp. Schematic created using http://wormweb.org. Middle, Wild-type and mutant nucleotide sequences spanning the mutagenesis site. The gRNA target site (red line) and restriction enzyme (RE) recognition site (green line) are labeled. Bottom, Amino acid sequence indicating that the *myrf^ue70^* mutation results in shift in the open reading frame leading to downstream coding for a premature stop codon (*). ***B***, The relative concentration of mbp mRNA is reduced by 95% in mutants (0.04 ± 0.03 au) compared with wild types (1.003 ± 0.13 au, *p* = 0.0002, unpaired *t* test, *N* = 3 adult brains per genotype). Error bars represent mean ± SD. ***C***, Transverse section of the spinal cord in an adult *myrf^ue70^* sibling showing extensive myelination of ventral spinal cord (dashed box). 20× objective. Scale bar: 100 µm. ***D***, TEM images of the spinal cord in the region of the ventral spinal tract (outlined in ***C***) in *myrf^ue70^* adult siblings (top) and mutants (bottom). Panels i–iv display different fields of view within the region of interest. Thick myelin sheaths are clearly visible in siblings, particularly surrounding the Mauthner axon. There is a lack of myelin surrounding the Mauthner axon in the mutant sample, and distinct reduction in the level of myelination in the remainder of surrounding spinal cord. Occasional hypomyelinated and dysmyelinated axons can be observed in the mutant samples. Scale bar: 5 µm (panels i–iii) and 1 µm (panel iv). m, Mauthner axon. ***E***, top, Brightfield images of *myrf^ue70^* wild-type and mutant larvae at 6 dpf. Black box defines the anatomic region imaged across animals. Scale bar: 0.5 mm. Bottom, Confocal microscopy images of the spinal cord at 6 dpf in *myrf^ue70^* Tg(mbp:eGFP-CAAX) larvae. Scale bar: 20 µm.

To begin our investigations into the role of CNS myelination in neural circuit function, we sought to establish a larval zebrafish model with CNS-specific hypomyelination. Mammalian studies have identified myelin gene regulatory factor (myrf) as a transcription factor vital for CNS myelin formation and maintenance (Emery et al., 2009; Bujalka et al., 2013). Zebrafish possess a single ortholog of myrf and, similar to mammals, myrf expression in the CNS appears to be restricted to oligodendrocytes (Treichel and Hines, 2018; Klingseisen et al., 2019). We used CRISPR/Cas9 technology to target a guide RNA to exon 2 of the zebrafish *myrf* gene, the first conserved exon across all predicted splice variant isoforms, and in doing so created the *myrf^ue70^* mutant (Materials and Methods; Fig. 1*A*). Morphometric analysis of larval body features of *myrf^ue70^* mutants at larval stages showed them to be indistinguishable from siblings (data not shown), and in contrast to mammalian myrf mutants (Emery et al., 2009), homozygous *myrf^ue70^* mutants remain viable through to adulthood. Adult *myrf^ue70^* mutants exhibited an almost complete absence of *mbp* mRNA (Fig. 1*B*), and TEM assessment indicated effectively no myelin in the adult spinal cord (Fig. 1*C*,*D*). In addition, and unlike larvae, homozygous adult *myrf^ue70^* were grossly identifiable from their siblings by their smaller size. Adult *myrf^ue70^* mutants were also infertile, because of the absence of detectable gonadal tissue in females, confirmed via histopathology, which also revealed evidence of cardiomyopathy (data not shown), findings consistent with proposed roles of myrf outside the CNS (Pinz et al., 2018; Hamanaka et al., 2019; Rossetti et al., 2019).

**Figure 2.**
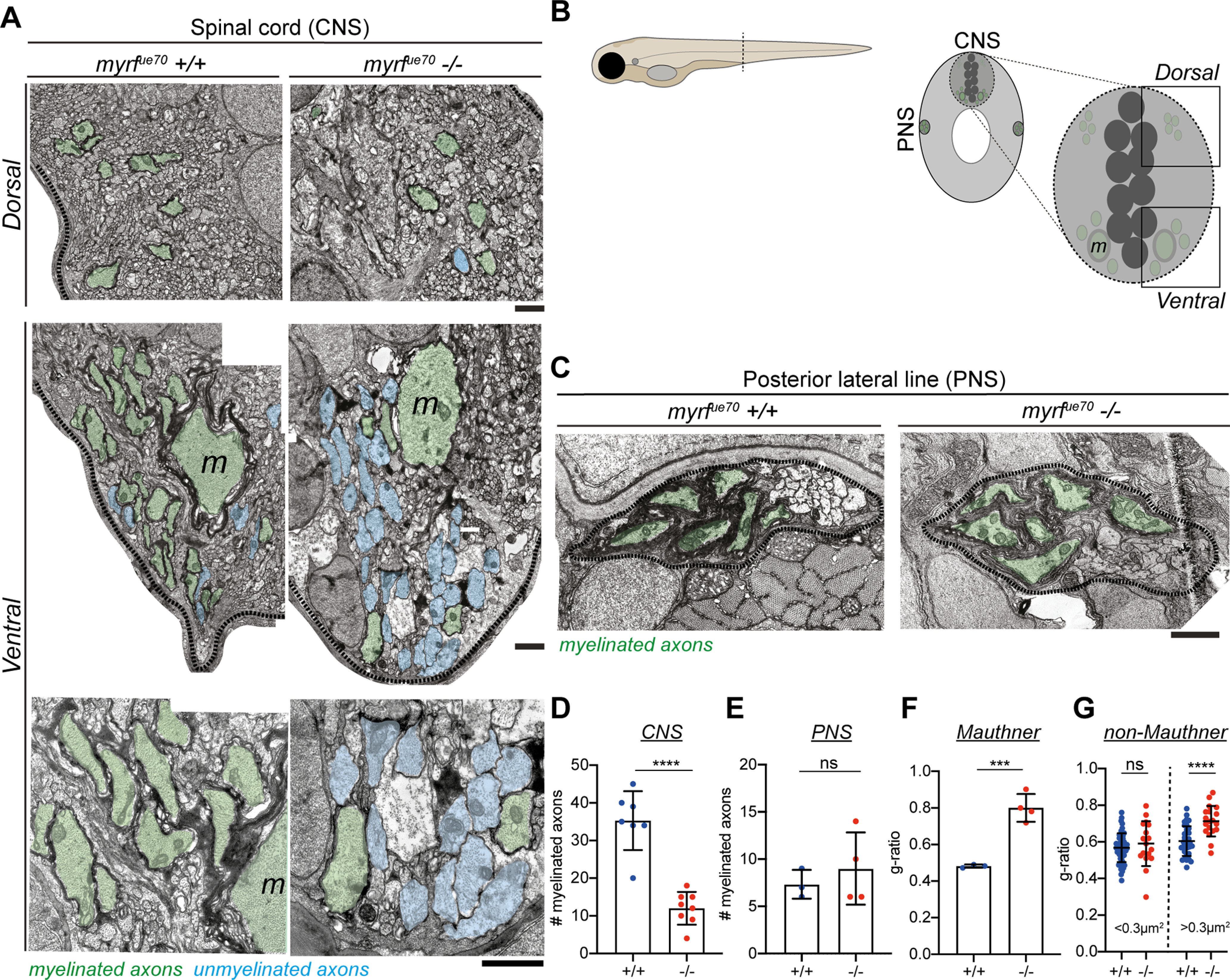
*myrf^ue70^* mutants display CNS-specific hypomyelination at 6 dpf. ***A***, TEM images of the myelinated tracts in the dorsal (top row) and ventral spinal cord (bottom rows). Scale bar: 1 µm. ***B***, Schematic of the transverse section of a 6 dpf larval zebrafish at the level of the urogenital opening. Inset, transverse section of the spinal cord at the same level. Myelinated (green) axons are located in the ventral and dorsal spinal tracts of the spinal cord (CNS) as well as the posterior lateral line (PNS). m, Mauthner axons. ***C***, TEM images of the posterior lateral line at 6 dpf. Scale bar: 1µm. ***D***, The average number of myelinated axons in one hemi-spinal cord is reduced by 66% in mutants (wild types: 35.29 ± 7.83 myelinated axons, mutants: 12.00 ± 4.34 myelinated axons, *p* ≤ 0.0001, unpaired *t* test, *N* = 7 wild types, *N* = 8 mutants). ***E***, The number of myelinated axons in the PNS is similar between genotypes (wild types: 7.33 ± 1.53 myelinated axons, mutants: 9.00 ±3.83 myelinated axons, *p* = 0.52, unpaired *t* test, *N* = 3 wild types, *N* = 4 mutants). ***F***, G-ratio of Mauthner axons in wild-type and mutant siblings (wild types: 0.48 ± 0.009, mutants: 0.80 ± 0.08, *p* = 0.0009, unpaired *t* test). For ***D***-***F***, error bars represent mean ± SD. ***G***, g-ratios for myelinated axons for small caliber (area <0.3 µm^2^) and large caliber (area >0.3 µm^2^) myelinated axons. The g-ratio of small caliber axons is similar between groups [wild types: 0.57 (0.52–0.62), mutants: 0.59 (0.52–0.70), *p* = 0.51, Mann–Whitney test, *n* = 53 myelinated axons in wild types, *n* = 17 myelinated axons in mutants error bars represent median and IQR]. The g-ratios for large caliber axons are significantly higher in mutants than wild-type siblings (wild types: 0.60 ± 0.08, mutants: 0.71 ±0.08, *p* ≤ 0.0001, unpaired *t* test, *n* = 33 myelinated axons in wild types, *n* = 19 myelinated axons in mutants error bars represent mean ± SD).

Given the potential to study myelination of well-defined circuits at high resolution over time at larval stages when *myrf^ue70^* mutants are morphologically indistinguishable from siblings, we next analyzed our transgenic reporter of myelination Tg(mbp:eGFP-CAAX) at 6 dpf. This indicated that the gross level of CNS myelination was also reduced in *myrf^ue70^* mutant larvae relative to wild-type siblings (Fig. 1*E*). To quantify myelination in larvae, TEM was performed on transverse sections of the spinal cord (CNS) and posterior lateral line nerve of the PNS at 6 dpf (Fig. 2*A–C*). At this time point, we observed a 66% reduction in the number of myelinated axons in the spinal cord of *myrf^ue70^* mutants relative to wild-type siblings (35.29±7.83 myelinated axons in wild types, 12.00 ±4.34 myelinated axons in mutants, *p* ≤ 0.0001, unpaired *t* test; Fig. 2*D*). In contrast, and demonstrating specificity of hypomyelination to the CNS, similar numbers of myelinated axons were observed in the PNS of mutant and wild-type siblings (7.33 ±1.53 myelinated axons in wild types, 9.00 ±3.83 myelinated axons in mutants, *p* = 0.52, unpaired *t* test; Fig. 2*E*).

Despite the large number of unmyelinated axons in *myrf^ue70^* mutants, our TEM analyses indicated that some axons remained ensheathed in the larval CNS, including the very large diameter Mauthner axons, the first RS axons to be myelinated in the zebrafish CNS (Almeida et al., 2011). Although Mauthner axons were ensheathed in *myrf^ue70^* mutants at 6 dpf, they had significantly thinner myelin sheaths compared with wild-type siblings (average g-ratio: 0.48 ±0.009 in wild types, 0.80 ±0.08 in homozygous mutants, *p* = 0.0009, unpaired *t* test; Fig. 2*F*). A similar finding was observed in the other axons that were ensheathed in *myrf^ue70^* mutants at this stage, with greater g-ratio values (denoting thinner myelin) for other large caliber (>0.3 µm^2^) axons in mutants than in wild-type siblings (average g-ratio: 0.60 ± 0.08 wild types, 0.71 ± 0.08 mutants, *p* ≤ 0.0001, unpaired *t* test; Fig. 2*G*). Despite the generally severe hypomyelination phenotype, the presence of some large caliber myelinated axons in zebrafish *myrf^ue70^* mutants at larval stages contrasts with our analysis of adult zebrafish mutants and myrf mutant mice which both have essentially a complete absence of CNS myelin (Emery et al., 2009). This suggests that the full effects of myrf knock-out may be masked at early stages, either by maternal gene expression or genetic compensatory mechanisms (Rossi et al., 2015).

**Figure 3.**
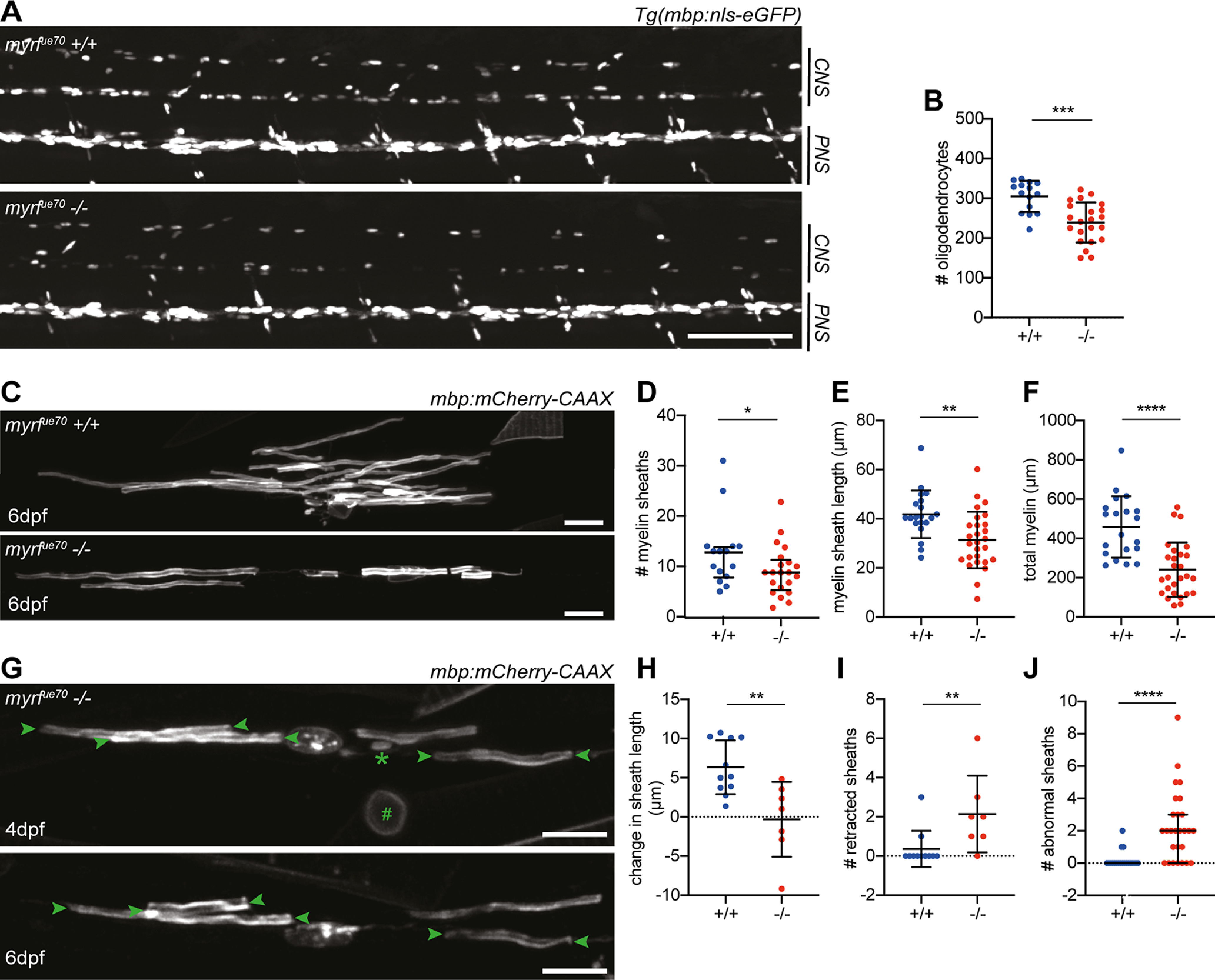
*myrf^ue70^* mutants have fewer oligodendrocytes which produce less myelin and fail to maintain myelin sheaths over time. ***A***, Confocal images of the spinal cord at 6 dpf in sibling control and *myrf^ue70^* Tg(mbp:nls-eGFP) larvae. Scale bar: 100 µm. ***B***, Oligodendrocyte numbers in the spinal cord at 6 dpf (wild-type: 304.8 ± 39.07, mutants: 239.3 ± 50.48, *p* = 0.0002, unpaired *t* test, *N* = 15 wild types, *N* = 22 mutants). Error bars represent mean ± SD. ***C***, Representative confocal images of single oligodendrocytes mosaically labeled with mbp:mCherry-CAAX reporter construct in a wild type (top) and mutant (bottom) at 6 dpf. Scale bar: 15 µm. ***D***, Average myelin sheath number was reduced in *myrf^ue70^* mutants relative to wild-type siblings at 6 dpf [wild types: 10.50 (7.00–14.00) sheaths per cell, mutants: 7.00 (5.00–10.50) sheaths per cell, *p* = 0.02, Mann–Whitney test]. Values and error bars represent median and IQR. ***E***, Average myelin sheath length was reduced from 41.83 ±9.68 µm in wild types to 31.35 ±11.49 µm in mutants at 6 dpf (*p* = 0.002, unpaired *t* test). Error bars represent mean ± SD. ***F***, Total myelin produced per oligodendrocyte was reduced from 458.2 ± 156.4 µm in wild types to 241.1 ±138.6 µm in mutants at 6 dpf (*p* ≤ 0.0001, unpaired *t* test). Error bars represent mean ± SD. ***D–F***, *N* = 20 wild types, *N* = 27 mutants. ***G***, Confocal images of a single mutant oligodendrocyte labeled with mbp:mCherry-CAAX at 4 and 6 dpf. A myelin sheath (*) and myelinated neuronal cell body (#) are observed at 4 dpf and subsequently retracted by 6 dpf. Arrowheads label myelin sheaths which are observed to shrink between 4 and 6 dpf. Scale bar: 15 µm. ***H***, Myelin sheaths belonging to wild-type oligodendrocytes demonstrated a net growth of 6.24 ± 3.43 µm between 4 and 6 dpf, while mutants display net shrinkage of myelin sheaths by −0.31 ± 4.79 µm (*p* = 0.003, unpaired *t* test). Error bars represent mean ± SD. ***I***, Between 4 and 6 dpf, wild-type oligodendrocytes retracted 0 (0–0) myelin sheaths, while mutants retracted 2 (1–3) myelin sheaths (*p* = 0.009, Mann–Whitney test). Error bars represent median and IQR. ***J***, Number of abnormal myelin sheaths at 6 dpf [wild types: 0.00 (0.00–0.00); mutants: 2 (0.00–3.00), *p* ≤ 0.0001, Mann–Whitney test]. Error bars represent median and IQR. ***H***, ***I***, *N* = 11 wild types, *N* = 7 mutants. ***J***, *N* = 20 wild types, *N* = 27 mutants.

To examine the cellular basis of CNS hypomyelination in *myrf^ue70^* mutant larvae, we first assessed myelinating oligodendrocyte number using the transgenic reporter Tg(mbp:nls-eGFP) (Karttunen et al., 2017; Fig. 3*A*). At 6 dpf, the time point at which TEM was performed, the number of detectable oligodendrocytes was reduced by 21% in *myrf^ue70^* mutants relative to wild-type siblings (*p* = 0.0002, unpaired *t* test; Fig. 3*B*). In addition, the fluorescent intensity of *myrf^ue70^* mutant oligodendrocyte nuclei was reduced, consistent with reduced *mbp* expression. Because, the reduction in cell number was not sufficient to explain the reduction in myelin observed using TEM, we assessed the morphology of individual myelinating oligodendrocytes using mosaic cell labeling with the mbp:mCherry-CAAX reporter construct (Almeida et al., 2011; Fig. 3*C*). We found that both myelin sheath number (*p* = 0.02, Mann–Whitney test; Fig. 3*D*) and length (*p* = 0.002, unpaired *t* test; Fig. 3*E*) were reduced in *myrf^ue70^* mutants by 33% and 25%, respectively, at 6 dpf, with total myelin (sum of sheath lengths) per individual oligodendrocyte reduced by 47% in mutants relative to wild types (*p* ≤ 0.0001, unpaired *t* test; Fig. 3*F*). In addition to being required for the initiation of myelination, previous studies in rodents indicate that myrf is also essential for myelin sheath maintenance (Koenning et al., 2012). Having observed that adult *myrf^ue70^* mutants have a much more severe hypomyelination phenotype than larvae (Fig. 1*D*), we wanted to assess how the morphology of single oligodendrocytes changed over time. To do so, we imaged single oligodendrocytes at 4 dpf and again at 6 dpf (Fig. 3*G*). We found that between these time points mutant oligodendrocytes demonstrated a net shrinkage in myelin sheath length, while wild-type oligodendrocytes showed a net growth (*p* = 0.009, Mann–Whitney test; Fig. 3*H*). Furthermore, the number of myelin sheaths that were completely retracted during this timeframe was significantly higher in *myrf^ue70^* mutant oligodendrocytes (*p* = 0.003, unpaired *t* test; Fig. 3*I*). Also consistent with a failure to maintain healthy myelin sheaths, the number of myelin sheaths exhibiting an abnormal morphology (i.e., incomplete wrapping, abnormal elongation profiles or myelin blebs) was significantly higher in mutant versus wild-type oligodendrocytes at 6 dpf (Fig. 3*J*).

In summary, disrupting myrf leads to a CNS-specific hypomyelination phenotype in larval zebrafish, caused by a reduction in the number of oligodendrocytes, with those that remain having fewer and shorter sheaths. The majority of sheaths that are made are thinner, and, based on our documentation of almost complete absence of myelin in adults, not maintained long-term. Therefore, the phenotype in the *myrf^ue70^* mutant fulfilled our aim to generate a CNS-specific model of hypomyelination to study the effects on neural circuit function at larval stages.

### *myrf^ue70^* mutants exhibit an increase in the latency to perform startle responses and an impaired behavioral choice in response to a defined auditory stimulus

**Figure 4.**
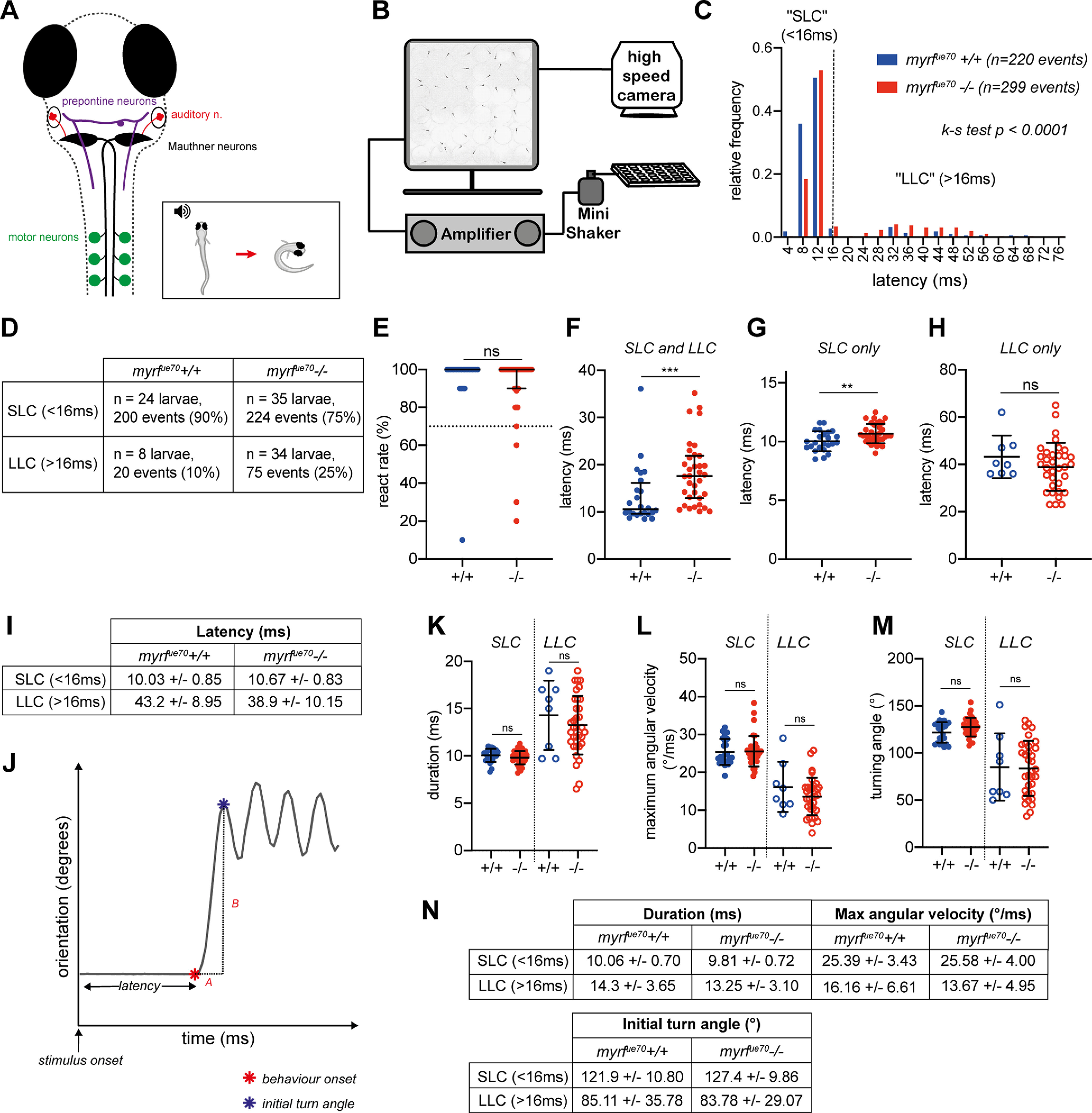
*myrf^ue70^* mutants exhibit increased latency to perform startle responses, and a tendency to perform avoidance behavior, in response to defined acoustic stimuli. ***A***, Overview of the neuronal circuitry involved in motor response to auditory stimuli. Startle response (SLC): sensory input from the ear, via the auditory nerve (red), is received at the lateral dendrite of the Mauthner cell body (black). The axon of the Mauthner cell crosses into the contralateral aspect of the spinal cord where it extends along the ventral tract to recruit motor neurons directly along the length of the larvae. Recruitment of motor neurons allows muscle contraction on the side of the body contralateral to the stimulus, allowing a rapid, high-velocity c-bend (motor response) away from the stimulus (inset). Avoidance behavior (LLC): sensory input is detected by prepontine neurons (purple) in the hindbrain, which recruit ipsilateral motor neurons indirectly, resulting in a low-velocity, longer latency, c-bend away from the stimulus. ***B***, Schematic of the behavioral rig. ***C***, Relative frequency histogram displaying the distribution of latencies for behavioral responses in response to acoustic stimuli in wild-type and mutant larvae (*N* = 24 wild-type larvae, *n* = 220 events; *N* = 35 mutant larvae, *n* = 299 events; Kolmogorov–Smirnov test, *p* ≤ 0.0001). ***D***, Number and proportion of events (SLC vs LLC) per genotype. ***E***, React rate per fish (median react rate = 100% in both wild types and mutants, *p* = 0.24, Mann–Whitney test, *N* = 25 wild-type larvae, *N* = 38 mutant larvae). Larvae are excluded from subsequent analysis if they exhibit a react rate <70%. ***F***, Average latency values per fish [wild type: 10.55 ms (9.6–16.15 ms), mutants: 17.6 ms (12.9–21.88 ms), *p* = 0.003, Mann–Whitney test]. ***G***, Average latency of SLC (<16 ms; wild types: 10.03 ± 0.85 ms, mutants: 10.67 ± 0.83 ms, *p* = 0.006, unpaired *t* test). ***H***, Average latency of LLC (>16 ms; wild types: 43.20 ± 8.95 ms; mutants: 38.91 ± 10.15 ms, *p* = 0.28, unpaired *t* test). ***I***, Mean and SDs values for SLC and LLC responses per genotype. ***J–M***, Analysis of c-bend kinematics. ***J***, Example trace of orientation over time during a behavioral response to an acoustic stimulus. C-bend kinematics are calculated from individual traces for each response per fish. Latency is the time from stimulus onset to behavioral onset (red star). C-bend duration (A) is time from behavior onset to initial turn angle (blue star). Maximum angular velocity is defined as the change in orientation over time (B/A). Turning angle equates to the initial turn angle. ***K***, Initial turn duration (SLC: wild types: 10.06 ± 0.70 ms, mutants: 9.81 ± 0.72 ms, *p* = 0.20, unpaired *t* test; LLC: wild types: 14.30 ± 3.65 ms, mutants: 13.25 ± 3.10, *p* = 0.42, unpaired *t* test). ***L*,** Maximum angular velocity [SLC: wild types: 24°/ms (22.78–28.68°/ms), mutants: 25°/ms (23.10–26.60°/ms), *p* = 0.73, Mann–Whitney test; LLC: wild types: 16.16 ± 6.61°/ms, mutants: 13.67 ± 4.95°/ms, *p* = 0.24, unpaired *t* test]. ***M***, Initial turn angle (SLC: wild types: 121.9 ± 10.80°, mutants: 127.4 ± 9.86°, *p* = 0.051, unpaired *t* test; LLC: wild types: 85.11 ± 35.78°, mutants: 83.78 ± 29.07°, *p* = 0.91, unpaired *t* test). ***N***, Descriptive statistics (mean ± SD) for c-bend kinematics. For ***G–H***, ***K–M***, SLCs, *N* = 24 wild types, *N* = 35 mutant larvae, LLCs, *N* = 8 wild-types, *N* = 34 mutant larvae. For ***E***, ***F***, ***L***, values represent median and interquartile range; for ***G–I***, ***K*** and ***M*** values represent mean ± SD.

Given that many larval zebrafish sensorimotor behaviors are mediated by RS neurons, whose axons are myelinated early and exhibit activity-regulated myelination (Koudelka et al., 2016), we hypothesized that *myrf^ue70^* mutants would display detectable differences in the performance of RS-mediated behaviors. To test this, we chose to first examine acoustic-startle behavior, for which the underlying circuit is relatively well described (Hale et al., 2016). Briefly, a high-intensity acoustic stimulus activates the auditory (VIIIth) nerve, which courses into the hindbrain to synapse onto the Mauthner cell at its lateral dendrite. Once the threshold potential is exceeded, an action potential is elicited and rapidly propagated along the Mauthner axon, which crosses into, and extends along, the contralateral tract of the spinal cord. Along its length, collateral branches make synapses with interneurons and primary motor neurons that coordinate motor output. Activation of a Mauthner axon results in a stereotypical, high-velocity “c-bend” away from the stimulus, followed by a fast burst swim (Kimmel et al., 1974; Zottoli et al., 1995; Fig. 4*A*). The latency to perform such a response is defined as the time taken from stimulus presentation to the onset of a c-bend (Fig. 4*J*). Given that myelin increases conduction velocity along a single axon (Waxman, 1980), we made the prediction that the latency to execute the motor responses following an acoustic stimulus would be delayed in *myrf^ue70^* mutants.

Motor behavior was assessed using an established high-throughput assay (Burgess and Granato, 2007a). *myrf^ue70^* larvae were arrayed into individual wells of a 6 × 6 custom made plate attached to an amplifier delivering a series of acoustic stimuli at 20-s intervals (Fig. 4*B*). Using a high-speed (1000 Hz) camera, behavioral responses were recorded and subsequently analyzed using FLOTE software (Burgess and Granato, 2007a,b). Overall, the frequency of responses to acoustic stimuli was similar between groups (Fig. 4*E*). However, on average, *myrf^ue70^* mutants exhibited a 66% increase in their average latency to elicit a response compared with wild-type siblings [wild types: 10.55 ms (9.6–16.16 ms), mutants: 17.60 ms (12.90–21.88 ms), *p* = 0.003, Mann–Whitney test; Fig. 4*F*].

Interestingly, larval behavioral responses to acoustic stimuli can be modulated across variable stimulus properties, exhibiting decision-making capabilities of the underlying circuitry (Burgess and Granato, 2007a; Jain et al., 2018). For example, in larval zebrafish, while high intensity threatening stimuli induce the short-latency c-bend startle response, also known as the SLC, lower stimulus intensities induce a distinct longer latency reorientation-like behavior, initially defined as an LLC. These kinematically and behaviorally distinct responses are executed by activity in partially overlapping circuitry, with the crucial difference that SLCs are driven by recruitment of Mauthner neurons, while LLCs appear to be driven by alternative pathways, e.g., prepontine neurons (Burgess and Granato, 2007a; Marquart et al., 2019; Fig. 4*A*). Given that hypomyelination in *myrf^ue70^* is widespread within the CNS, we anticipated that the large overall increase in latency to respond to acoustic stimuli might be because of significant delays in the performance of both SLC and LLC responses. However, when data were segmented into SLCs or LLCs, the latency to perform an SLC was increased by 6.4% (10.03 ± 0.85 ms in wild types, 10.67 ± 0.83 ms in mutants, *p* = 0.006, unpaired *t* test; Fig. 4*G*,*I*), but the latency to perform LLCs remained unaffected (Fig. 4*H*,*I*), begging the question as to what caused the much larger overall increase in latencies to respond to acoustic stimuli.

We reasoned that if the latency to perform SLCs was only affected to a small degree and LLCs not at all, the overall large increase in latency to perform all responses might be because of a biased selection of the longer latency LLCs over the much shorter latency SLCs. Indeed, when we compared their relative frequency, we saw that LLCs represented a significantly increased proportion of behavioral responses in *myrf^ue70^* mutants relative to wild types (SLC:LLC ratio, 10:1 in wild types and 2.9:1 in mutants, *p* ≤ 0.0001, Kolmogorov–Smirnov test; Fig. 4*C*,*D*). To ensure that this apparent bias in behavioral selection was not because of SLCs simply being so slow as to be detected as LLCs, we analyzed additional kinematic parameters (Fig. 4*J–N*), which have specific values associated with each type of response (Burgess and Granato, 2007a). No differences were found in the duration, maximum angular velocity or initial turning angle of SLCs or LLCs between wild-type and mutant larvae (Fig. 4*K–M*), consistent with the conclusion that the increased frequency of LLCs represents true LLC events, rather than delayed and inappropriately classified SLCs.

In summary, we have shown that *myrf^ue70^* mutants exhibited delayed latency to perform Mauthner-mediated startle responses (SLCs), and an unexpected bias toward performing Mauthner-independent reorientation behaviors (LLCs) in response to the same acoustic stimuli. This shows that hypomyelination in the larval zebrafish can be detected in overt changes to behavior and highlights the complexity of how dysregulation of myelination impacts circuit function, even when executing relatively simple sensorimotor transformations.

### Action potential conduction is impaired along the Mauthner axon in *myrf^ue70^* mutants

**Figure 5.**
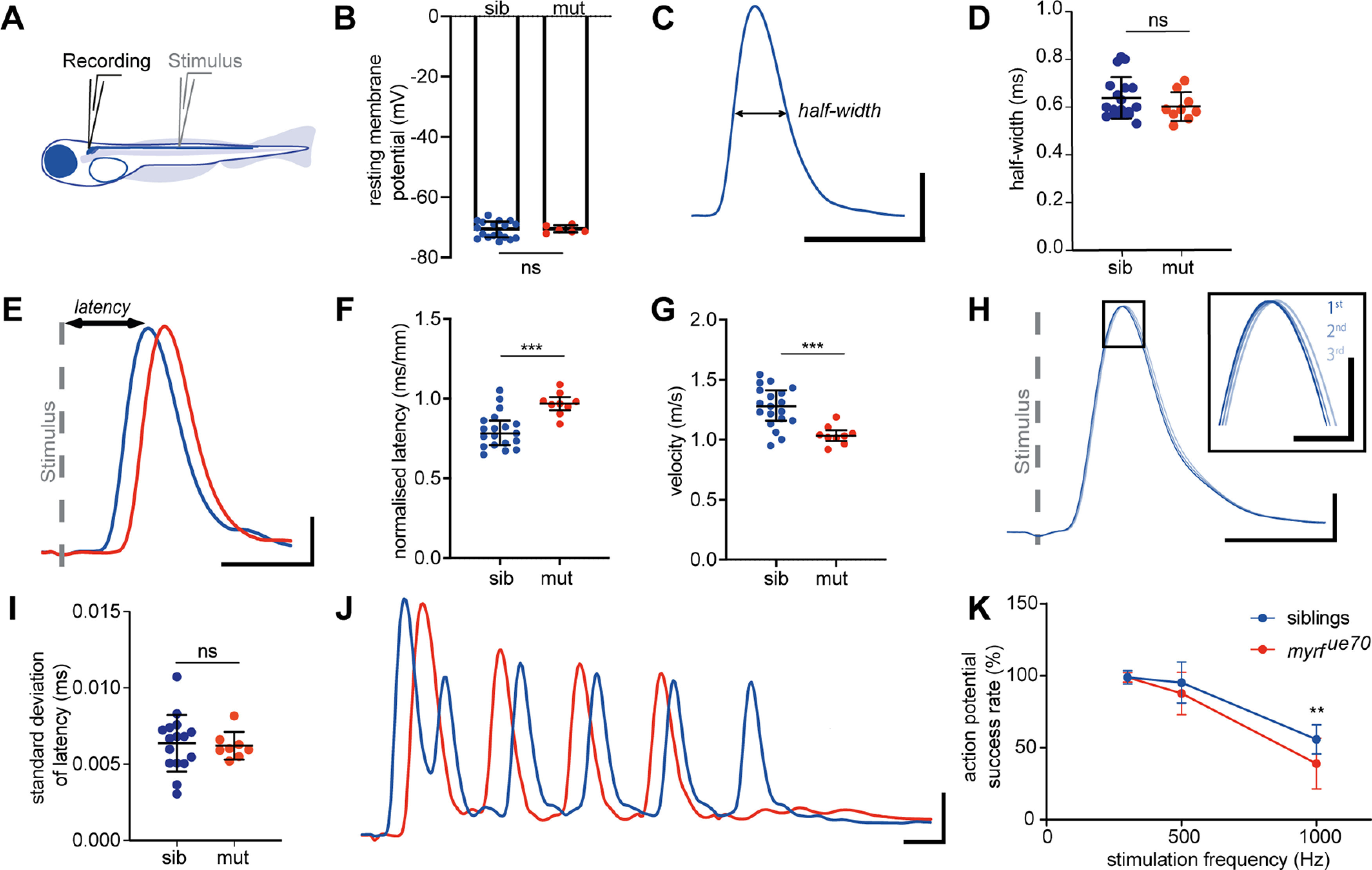
Whole cell current-clamp recordings from Mauthner cells demonstrate slower conduction velocity times and abnormal spiking profiles in *myrf^ue70^* mutants. ***A***, Electrophysiological preparation for recording from Mauthner neuron in a whole-cell current clamp configuration while stimulating with an extracellular monopolar field electrode midway through the spinal cord. ***B***, Resting membrane potential is unchanged in siblings (*n* = 18 cells): −70.82 ± 2.76 mV, mutants (*n* = 6 cells): −70.68 ± 1.25 mV, *p* = 0.9077 at 6 dpf. ***C***, Sample trace of an action potential recorded at 6 dpf in a wild-type fish illustrating the measurement of half-width. Half-width is described as width of action potential (ms) at its half height. ***D***, Half-width of action potential is unchanged [siblings (*n* = 18 cells): 0.64 ± 0.09 ms, mutants (*n* = 9 cells): 0.60 ± 0.06 ms, *p* = 0.2610 at 6 dpf]. ***E***, An example of current–clamp recording from Mauthner neuron in a 6 dpf wild type and mutant following field stimulation (stimulus artifact is indicated by a gray dashed line). Latency is described as time from the onset of stimulus artifact to the peak of action potential. ***F***, Normalized action potential latency is increased in mutants at 6 dpf [siblings (*n* = 19 cells): 0.80 ± 0.11 ms/mm, mutants (*n* = 9 cells): 0.97 ± 0.07 ms/mm, *p* = 0.0003 at 6 dpf]. ***G***, Conduction velocity of Mauthner action potentials is significantly decreased in mutant larvae [siblings (*n* = 19 cells): 1.27 ± 0.17 m/s, mutants (*n* = 9 cells): 1.04 ± 0.08 m/s, p 0.0005 at 6 dpf]. ***H***, Sample traces of three subsequent action potentials recorded from the same wild-type Mauthner cell at 6 dpf superimposed and aligned to the peak of stimulus artifact. The area outlined by the rectangle is magnified in the inset and demonstrates slight imprecision of action potential arrival. ***I***, Precision of action potential arrival is comparable in siblings and mutants [siblings (*n* = 16 cells): 0.0064 ± 0.0019 ms, mutants (*n* = 8 cells): 0.0062 ± 0.0009 ms, *p* = 0.8166 at 6 dpf]. ***J***, Sample trace of a train of action potentials fired following 10 stimuli at 1000 Hz at 6 dpf in a *myrf^ue70^* mutant and sibling. ***K***, Mauthner neurons in mutant larvae do not sustain prolonged action potential trains of high-frequency stimulation [siblings (*n* = 19 cells): 55.79 ± 10.17% mutants (*n* = 9 cells): 38.89 ± 17.64% at 6 dpf, *p* = 0.0014 at 6 dpf]. For ***B***, ***D***, ***F***, ***G***, ***I***, ***K***, error bars represent mean ± SD. Unpaired *t* test for ***B***, ***D***, ***F***, ***G***, ***I*** and a two-way ANOVA for ***K***. Scale bars: 10 mV and 1 ms (***C***, ***E***, ***H***, ***J*)** and 5 mV and 200 μs (***H***, inset).

In order to investigate how myelination affects conduction along larval zebrafish axons, we set out to establish an electrophysiological platform that would allow us to measure and compare multiple aspects of axonal conduction *in vivo*. We focused our analysis on the Mauthner neuron and axon, because of its characteristic morphology and anatomic location, and given its established role in mediating the SLC. To begin with, we performed whole-cell current-clamp recordings of the Mauthner neuron cell body while stimulating its axon in the spinal cord with an extracellular electrode (Fig. 5*A*). We first tested whether loss of myrf function affected intrinsic properties of the Mauthner neuron, by assessing its resting membrane potential: we found that this remained stable in mutants (siblings: −70.82 ± 2.76 mV, mutants: −70.68 ± 1.25 mV, *p* = 0.9077, unpaired *t* test**;** Fig. 5*B*). Our experimental configuration allowed us to record antidromic action potentials propagating along the Mauthner axon. Therefore, we next assessed whether the shape of action potentials was disrupted by hypomyelination, by measuring the width of the action potential at its half-height (action potential half-width) at 6 dpf, which we found to be similar in control and *myrf^ue70^* mutant animals (siblings: 0.64 ± 0.09 ms, *myrf^ue70^* mutants: 0.60 ± 0.06 ms, *p* = 0.2610, unpaired *t* test; Fig. 5*C*,*D*). These data indicate that the degree of hypomyelination along Mauthner axons in *myrf^ue70^* mutants at these stages does not affect the Mauthner resting membrane potential or greatly affect the shape of the action potentials.

Given the well-defined role for myelin in speeding-up action potential conduction, and the evidence of an increased latency to perform the Mauthner-dependent SLC response, we next measured the latency of action potential conduction along the Mauthner axon in controls and *myrf^ue70^* mutants. This analysis showed that the normalized latency of action potentials was significantly increased in *myrf^ue70^* mutants when compared with siblings (siblings: 0.80 ± 0.11 ms/mm, mutants: 0.97 ± 0.07 ms/mm, *p* = 0.0003; Fig. 5*F*) resulting in an 18% reduction in conduction velocity (siblings: 1.27 ± 0.17 m/s, mutants: 1.04 ± 0.08 m/s, *p* = 0.0005; Fig. 5*G*). This reduction in conduction velocity supports our finding of a delayed execution of SLCs in *myrf^ue70^* mutants. We next assessed whether the precision of action potential propagation might be impaired because of hypomyelination, which might interfere with synaptic signaling in the circuit. To do so, we measured the “jitter,” or imprecision, in the timing of action potential arrival following stimulation, as the SD of 30 action potential peak times aligned to the stimulus artifact (Fig. 5*H*). No differences were observed in the precision of action potential arrival in *myrf^ue70^* mutants at 6 dpf (siblings: 0.006 ± 0.002 ms, mutants: 0.006 ± 0.0009 ms, *p* = 0.8166, unpaired *t* test, Fig. 5*I*). These data suggest that hypomyelination leads to slower, but nonetheless precise, action potential propagation.

Given that the action potentials conducted along Mauthner axons in *myrf* mutants are likely to be sufficient to trigger downstream motor output, albeit with a longer delay, we next asked whether the hypomyelination of Mauthner axon might lead to an increased failure to reliably propagate action potentials. Therefore, we implemented a strategy to robustly test the ability of the myelinated axon to faithfully transmit action potentials. With our preparation, we observed that the Mauthner cell could spontaneously fire short trains of action potentials (1–10) at high frequency (∼300Hz) while in the resting state, before adding pharmacological reagents to block network-level input on to Mauthner and ahead of taking control of stimulating activity in the preparation, for the analyses noted above (data not shown). On the basis of this observation, and given the evidence from studies in rats that dysmyelination can influence firing frequency (Kim et al., 2013) we established a high-frequency stimulation paradigm to assess how hypomyelination affected the ability of the Mauthner axon to sustain high-frequency firing of action potentials. To do so, we used our field stimulation procedure and delivered 10 stimuli at various frequencies via the stimulating electrode and recorded the number of action potentials fired by the Mauthner cell, which allowed us to assess action potential success rate (Fig. 5*J*). Given that myelination reduces axonal current leakage, we predicted that our high-frequency stimulation protocol may reveal failed action potential propagation. When we analyzed the success rate of action potential firing, we found that this was indistinguishable between siblings and mutants at 300 Hz, insignificantly different at 500 Hz, but significantly impaired at 1000 Hz, stimulation, where we found that Mauthner cells from mutants fired with a significantly lower success rate (siblings: 55.79 ± 10.17%, mutants: 38.89 ± 17.64%, *p* = 0.0014, two-way ANOVA; Fig. 5*K*). This assay suggests that hypomyelination impairs the ability of axons to propagate action potentials faithfully, which could contribute to the behavioral shift away from Mauthner-mediated responses to auditory stimuli.

In conclusion, we have established an electrophysiology platform that allows direct measurement of single cell (i.e., Mauthner) conduction properties *in vivo*. In doing so, we have demonstrated that hypomyelination of the Mauthner axon leads to slowed conduction velocity, and with a high-frequency stimulation paradigm we reveal a loss of fidelity of action potential propagation along the hypomyelinated Mauthner axon.

## Discussion

We have demonstrated that CNS hypomyelination leads to behavioral alterations and impaired conduction along axons in larval zebrafish. We found that *myrf^ue70^* mutant larvae exhibit CNS-specific hypomyelination, representing the first zebrafish model with which one can study the role of CNS myelin in behavior. These mutants exhibited an increased latency to execute the stereotypical rapid acoustic startle responses (SLCs) and were also biased toward performing longer latency reorientation behaviors (LLCs) in response to startle-inducing acoustic stimuli. The fact that our analysis revealed phenotypes in both the speed of executing a specific behavior and in the selection of the correct behavioral response to a sensory stimulus indicates the complex roles that myelination plays in regulating circuit function. These findings provide encouragement that studying additional behaviors will offer further entry-points into studying how alterations to myelination affect the function of other neural circuits. Indeed, there are now a large number of behavioral paradigms that allow analysis of larval zebrafish circuit function, from various sensorimotor transformations (Dunn et al., 2016; Naumann et al., 2016; Henriques et al., 2019), behaviors regulated by sensory experience over time (Burgess and Granato, 2007a; Wolman et al., 2011) and those driven by interindividual interactions, such as sociability (Dreosti et al., 2015; Larsch and Baier, 2018).

In addition to studying behavior, we established electrophysiological protocols to assess the conduction properties of single neurons and axons, focusing on the Mauthner neuron because of the ease of its identification and its involvement in the acoustic startle response. We found that conduction velocity along the hypomyelinated Mauthner axon was reduced, and that Mauthner axons in *myrf^ue70^* mutants exhibited an increased failure to propagate action potentials in response to high-frequency stimulation. It remains to be determined precisely how disruption to the conduction properties of neurons and axons caused by hypomyelination affects circuit function and behavioral outputs. For example, the slowed execution of the SLC may be because of more than the slower conduction along the hypomyelinated Mauthner axon of *myrf* mutants, including slower conduction elsewhere in the circuit. Precisely how hypomyelination leads to a biased recruitment of LLCs over SLCs in response to the same auditory cue in *myrf^ue70^* mutants also remains to be elucidated, but could be influenced by the impaired ability to sustain high-frequency firing along the hypomyelinated Mauthner axons, and dysregulated recruitment of downstream motor pools. However, with our antidromic preparation, we cannot rule out the possibility that the reduced success rate of high-frequency action potential conduction was influenced by impaired generation of action potentials in the axon. Therefore, establishing methods to record orthodromic action potentials remains an important challenge for the future. In addition, to study how dysregulation of myelination influences synaptic signaling, electrophysiological analyses through the paired recordings of neurons known to communicate within circuits will be required. These studies, alongside the ability to assess the conduction properties of additional neurons, will be required to generate complete circuit models of how myelin influences even simple behaviors. Our study documented behavioral alteration and disruption to conduction in larvae with CNS-specific hypomyelination, but many challenges remain in integrating our understanding of circuit function across scales from conduction and synaptic communication through population-level neuronal activity and the execution of specific behaviors. However, we believe that the zebrafish represents a model in which such a multi-scale analyses of myelination on neural circuit function is feasible.

The larval zebrafish has numerous advantages that facilitate analyses of circuit function across scales. The larval CNS is relatively simple compared with mammalian models; with approximately one hundred thousand neurons by 6 dpf, only a relatively small proportion of which (on the order of a few hundred neurons) have myelinated axons at this stage (Hildebrand et al., 2017). The myelination of those axons is generally very stereotyped, with myelination of certain neuronal subtypes (e.g., RS neurons) adaptable and responsive to neuronal activity (Koudelka et al., 2016). With the aim of studying myelination from the perspective of neural circuits, we previously developed tools to study patterns of myelination along single axons *in vivo* (Koudelka et al., 2016). These tools, together with increasing availability of neuron-specific drivers coupled with circuit maps of the larval fish brain provide a great opportunity to map myelination at single cell resolution across the larval zebrafish CNS, and to do so over time. Even with myelination patterns mapped, a corresponding challenge will be to manipulate myelin from the point of view of specific neurons/axons and circuits. As noted above, it remains unclear whether the longer latency to execute the startle response is simply because of hypomyelination of Mauthner axons, or elsewhere in the circuit, and it may even be influenced by complex integrative functions that affect timing across the circuit. Therefore, it will be important to develop methods to regulate myelination in a neuron/axon and circuit-specific manner. One possibility might be to selectively ablate oligodendrocytes in specific circuits. Although oligodendrocyte ablation can be conducted at single cell resolution in zebrafish larvae (Auer et al., 2018), it leads to inflammatory reactions by cells such as microglia (Karttunen et al., 2017), which may be relevant to disease contexts, but would confound the disentangling of the role of myelin per se in healthy circuits. Therefore, an additional approach might be to express cell surface proteins that inhibit myelination (Redmond et al., 2016) along the axons of specific neuronal cell types (Burgess et al., 2009; Yamanaka et al., 2013; Tabor et al., 2018), selectively preventing their myelination. Furthermore, as signals and receptors that influence adaptive activity-regulated myelination are identified, yet more strategies to influence myelination in localized manners may emerge.

In addition to needing more refined methods to map and manipulate myelination of specific circuits, additional tools to assess function across scales from single axon to behaving animal will be required. Given the challenges of integrating complex electrophysiological protocols with behavioral observation in small zebrafish larvae, it is possible that optical methods will provide a better opportunity to bridge analyses across scales. Indeed, optical imaging approaches have already proven hugely powerful in the study of larval zebrafish brain function. For example, two-photon and light-sheet microscopy-based imaging studies allow the analysis of the activity of individual neurons (Abdelfattah et al., 2019) through to sampling the activity of effectively all neurons in the entire larval zebrafish brain, at multiple volumes per second with subcellular resolution (Ahrens et al., 2012, 2013; Chen et al., 2018). In fact, sophisticated imaging platforms that allow monitoring of neuronal activity in the brain during the execution of behaviors have been developed, including during acoustic stimulus-driven responses (Lacoste et al., 2015; Jain et al., 2018). Furthermore, the coordinated activity of ensembles of neurons have been investigated in the larval brain, which provides an opportunity to investigate how potentially even subtle alterations to myelination in development, health or disease might influence relatively high-order network activity (Sumbre et al., 2008; Romano et al., 2015; Wolf et al., 2017; Diana et al., 2019). To date, most optical analyses of neuronal activity in zebrafish have been conducted using genetically encoded Ca^2+^ reporters, but the limited temporal kinetics of even the fastest Ca^2+^ reporters may preclude the analysis of millisecond-scale changes to conduction properties, which our data indicate can be expected with disruption to larval myelination. However, ongoing development and refinement of voltage indicators appear to exhibit photodynamic properties with the sensitivity to detect functional changes to conduction and synaptic properties at the appropriate temporal resolution, including in larval zebrafish (Abdelfattah et al., 2019). Employing indicators that allow bona fide assessment of conduction in the intact brain, during the execution of behaviors has the potential to provide a transformative capacity to interrogate how myelin influences circuit function.

In summary, our study presents larval zebrafish as a viable model to study myelination across scales from molecular and cellular analyses of how myelin organizes and supports axons through to functional assessments of conduction, synaptic communication, network function, and behavior over time.
